# AttR2U-Net: A Fully Automated Model for MRI Nasopharyngeal Carcinoma Segmentation Based on Spatial Attention and Residual Recurrent Convolution

**DOI:** 10.3389/fonc.2021.816672

**Published:** 2022-01-28

**Authors:** Jiajing Zhang, Lin Gu, Guanghui Han, Xiujian Liu

**Affiliations:** ^1^ School of Biomedical Engineering, Sun Yat-sen University, Shenzhen, China; ^2^ RIKEN Center for Advanced Intelligence Project (AIP), Tokyo, Japan; ^3^ School of Information Engineering, North China University of Water Resources and Electric Power, Zhengzhou, China

**Keywords:** nasopharyngeal carcinoma, tumor segmentation, deep learning, spatial attention, recurrent convolution, residual connection

## Abstract

Radiotherapy is an essential method for treating nasopharyngeal carcinoma (NPC), and the segmentation of NPC is a crucial process affecting the treatment. However, manual segmentation of NPC is inefficient. Besides, the segmentation results of different doctors might vary considerably. To improve the efficiency and the consistency of NPC segmentation, we propose a novel AttR2U-Net model which automatically and accurately segments nasopharyngeal carcinoma from MRI images. This model is based on the classic U-Net and incorporates advanced mechanisms such as spatial attention, residual connection, recurrent convolution, and normalization to improve the segmentation performance. Our model features recurrent convolution and residual connections in each layer to improve its ability to extract details. Moreover, spatial attention is fused into the network by skip connections to pinpoint cancer areas more accurately. Our model achieves a DSC value of 0.816 on the NPC segmentation task and obtains the best performance compared with six other state-of-the-art image segmentation models.

## 1 Introduction

Nasopharyngeal cancer is a common malignant tumor occurring in the top and sidewalls of the nasopharyngeal cavity, with 833,019 new cases of nasopharyngeal cancer and 468,745 deaths in China alone during 2015 (1). Nasopharyngeal cancer affects a wide range of areas, from the nasal cavity forward to the conus, down to the oropharynx, and up to the skull. Moreover, it is mainly located in the central part of the head (2), making it difficult to treat with common surgical treatments. As such, the current mainstream treatment is radiotherapy. The lesion segmentation is one of the most critical factors affecting the effectiveness of radiotherapy.

**Figure 1:**
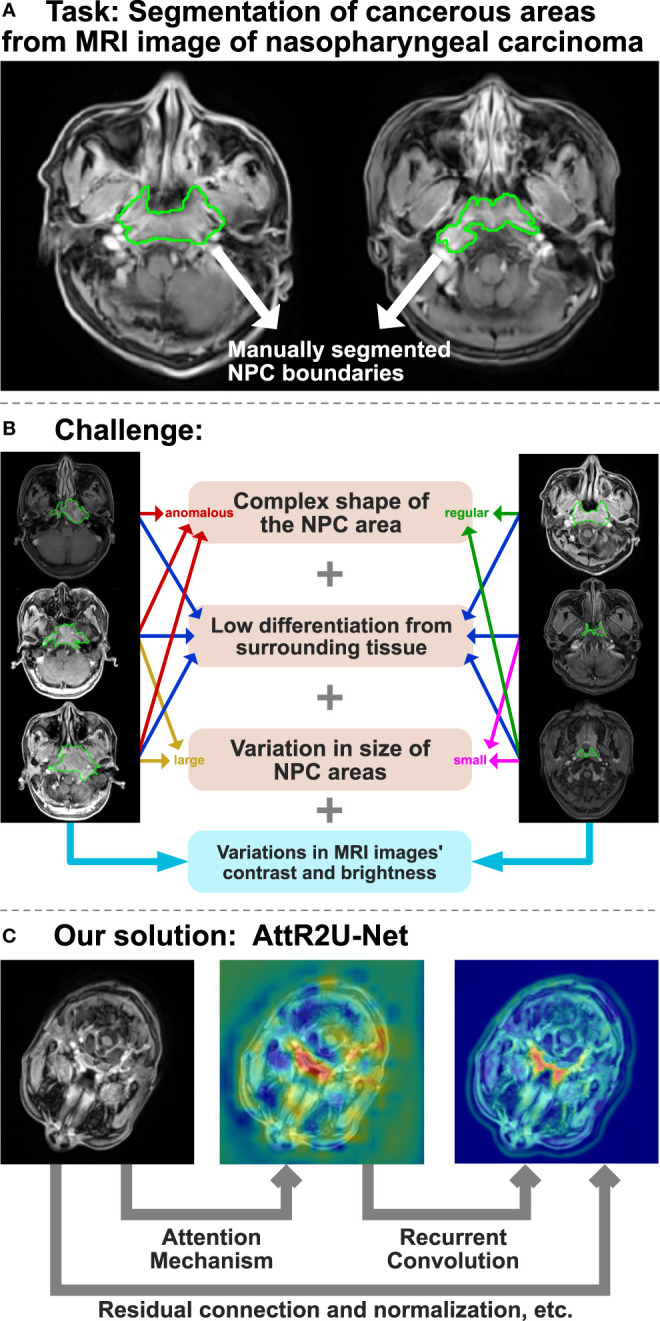
The overview of AttR2U-Net model for automatic segmentation of nasopharyngeal carcinoma. **(A)** The task of the AttR2U-Net model. **(B)** The challenges of segmentation arise from two main aspects, in which orange represents NPC internal causes and blue represents external cause. **(C)** The four main mechanisms of our solution: attention mechanism to localize NPC accurately, recurrent convolution to extract boundary details precisely, residual connection to build a deeper and more efficient model, and normalization to reduce the variations in MRI images.

However, traditional manual segmentation (**Figure 1A**) has three main drawbacks. First, the segmentation still currently relies on specialized physicians to manually segment nasopharyngeal carcinoma. However, manual segmentation is very time-consuming, taking at least 3 hour to complete the segmentation of one single patient (3, 4). Second, as the radiotherapy process progresses, the area of nasopharyngeal cancer keeps changing, making it necessary to re-identify the lesion. This undoubtedly increases the burden of doctors. Third, the effectiveness and quality of manual segmentation may vary significantly among physicians. Studies have shown that the segmentation area of nasopharyngeal cancer might vary from one physician to another for the same case (5, 6). The disagreement among physicians increases the complexity and uncertainty of the treatment. Thus, there is an urgent need for an automated and efficient method for accurate nasopharyngeal carcinoma segmentation.

However, automatic segmentation of nasopharyngeal carcinoma faces challenges from two main aspects, as shown in **Figure 1B**. First, the shape and size of the cancerous area are both very complex (7). Because of its invasive and aggressive nature (8), it can spread to the parapharynx, skull base, and even intracranial region. The size and shape of the cancerous area can vary considerably from patient to patient or from one patient’s different stages of the disease. Moreover, nasopharyngeal cancer occurs primarily in and around the nasopharynx, in the center of the head. The MR imaging of nasopharyngeal carcinoma may include a wide range of tissues near the cancerous area, such as the pharyngeal recess, pharyngeal orifice, pharyngeal tonsils, sphenoidal sinus, superior, middle, and inferior nasal concha, and hard and soft palate. Therefore, it is hard to distinguish nasopharyngeal cancer from these peripheral tissues, even for specialized doctors. Second, unlike most computer vision tasks with similar image quality (9, 10), the MR imaging quality may vary significantly for the same nasopharyngeal cancer. The variation of the image can be a result of various factors (1). MR imaging of nasopharyngeal cancer is highly dependent on imaging equipment (2). The imaging quality of the same equipment can be affected by the differences in physician’s operation, contrast agent variation, and patient status.

In the last decade, deep learning methods have been increasingly used in computer vision, such as target detection (11, 12), salient object detection (13, 14), video analysis (15, 16), and semantic segmentation (17), especially medical image segmentation (18). For example, Dey et al. propose a hybrid cascaded neural network for liver lesion segmentation (19), Singh et al. use a generative adversarial and convolutional neural network to achieve breast tumor segmentation in mammograms (20), Conze et al. use cascaded convolutional and adversarial deep networks to achieve abdominal multiorgan segmentation (21) and use deep convolutional encoder–decoders to achieve shoulder muscle MRI segmentation (22). Many studies show that deep learning methods can replace manual segmentation in medical image segmentation tasks and achieve satisfactory segmentation results while saving time and costs.

The most widely used structure among image segmentation models is the U-Net (23). It consists of an encoding module and a decoding module. The encoding module uses deep convolutional neural networks to gradually extract the features of the input image from local to global. The decoding module uses upsampling to recover the image resolution. Moreover, the results of each decoding layer are fused with the results obtained from the encoding layer by skip connection. However, experiments find that the ordinary U-net structure seems incompetent to segment complex tumors as nasopharyngeal carcinoma. It frequently fails to determine the location of nasopharyngeal cancer, especially when the cancerous area exists only in one side of the patient’s head. In addition, the model tends to include the surrounding tissues with similar brightness in the identified cancerous area. As such, the results of previous studies that use the classical U-Net structure to segment nasopharyngeal carcinoma have relatively low precision rates in NPC segmentation tasks. For example, Li et al. (24) only achieve a DSC value of 0.74.

Based on the U-Net structure, we propose AttR2U-Net and integrate several advanced deep learning method (**Figure 1C**), including spatial attention, residual connection, recurrent convolution, and normalization. Compared with general U-Net models, our method is novel with the following four main advantages:

- Our model introduces the attention mechanism so that it can learn the importance of features at different spatial locations (25).
- Our model uses the recurrent convolution (26) instead of the standard convolution to enhance the feature extraction capability.
- Our model adds residual connections to the neural network. It allows us to efficiently train deeper networks and solve the network degradation problem (27).
- Our model applies normalization to all the input. It solves the problem of contrast differences between different MRI images.

## 2 ATTR2U-Net for NPC Segmentation

**Figure 2:**
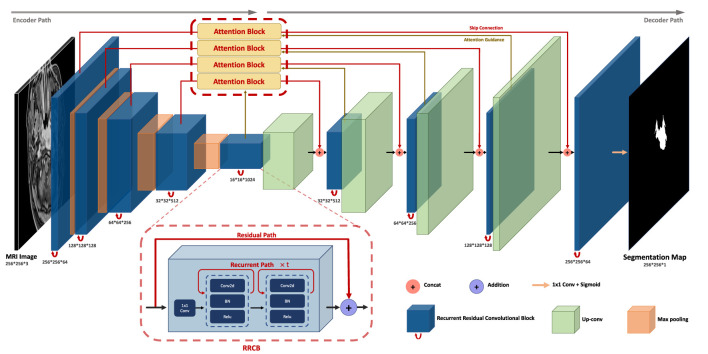
The architecture of AttR2U-Net: The encoder in AttR2U-Net consists of five Recurrent Residual Convolutional Blocks (RRCB). Each RRCB consists of a 1×1 convolution and two Recurrent Convolutional Layers, which loop Conv2d+BN+ReLU t times. Our model adds the input and output of each RRCN to achieve the residual connection. After each RRCB layer, our model sets a 2 × 2 max pooling layer to reduce the scale of the image by half. The decoder is symmetrically composed of four RRCBs, which are respectively connected with the first, second, third, and fourth encoding layer through the attention mechanism. The attention map is fed to the RRCB after concatenating with the upsampling output. After each RRCB, our model places an Up-Conv layer, including an upsampling layer and a Conv2d+BN+ReLU operation. Then output the segmentation result after a 1 × 1 convolution and a sigmoid layer.

The AttR2U-Net model proposed is shown in **Figure 2**. We incorporate three advanced computer vision methods, spatial attention, recurrent convolution, and residual connection, into the general U-Net through a sophisticated structural design.

The AttR2U-Net model consists of two parts: an encoder and a decoder. It first normalizes the input nasopharyngeal carcinoma image to solve the problem of contrast differences between MRI images, improving the model’s efficiency. The encoder with the residual connection and recurrent convolution gradually reduces the image’s resolution. The decoder implements upsampling through deconvolution with residual connection and recurrent convolution. The encoder can accurately extract local low-level and global high-level semantic information layer by layer, whereas the decoder recovers the high-resolution nasopharyngeal carcinoma segmentation map.

Additionally, by incorporating spatial attention in the feature fusion process, our model fuses the feature map from each layer of the encoder with the feature map from the decoder at the corresponding scales after passing through the attention block. Thus, our model can preserve both global macro-semantic information and local micro-detail in the upsampling process. Finally, the decoding module upsamples the feature map to the exact resolution as the input image and outputs it as the segmentation map.

### 2.1 Attention Mechanism

The attention mechanism aims to extract the different importance of each part of the input content, so that the model can focus on the needed parts of the input and ignore the less relevant parts. DeepMind firstly proposes attention for image classification (28). Then, it is widely used in various deep learning fields in recent years, including natural language processing (29, 30), image classification (31), video classification (32), and emotion recognition (33). Moreover, there are continuous studies to apply attention in deep convolutional neural networks (34). It results in a number of studies using attention for image segmentation, such as DANet (35) which applies self-attention on the spatial domain and channel domain, OCNet (36) which applies a semantic aggregation strategy, and PANet (37) which applies pyramidal structure attention on layer domain.

Inspired by Attention U-Net (38), our model implements spatial attention by adding attention blocks to the general U-net structure for the high-precision nasopharyngeal carcinoma segmentation.

Our model inserts the attention block in the skip connection connecting the encoder and decoder. The input of each attention block is the feature map *X_e_*_(i)_ output from the encoder on the corresponding downsampling scale and the feature map *X_d_*_(_ *_i-_*_1)_ output from the upper layer in the decoder. The attention block first linearly transforms them using channel-wise 1×1 convolutions with the parameters of *W_e_*_(_ *_i_*_)_ and *W_d_*_(_ *_i-_*_1)_, where *W_e_*_(_ *_i_*_)_ represents the parameters of the convolution to the input from the encoder and *W_d_*_(_ *_i-_*_1)_ represents the parameters of the convolution to the attention guidance from the decoder. Our model calculates the attention coefficients *α_i_* as


(1) $$\lambda_{i}=W_{e(i)}^{T}X_{e(i)}+W_{d(i-1)}^{T}X_{d(i-1)}+b_{\lambda_{i}}$$

**Figure 3:**
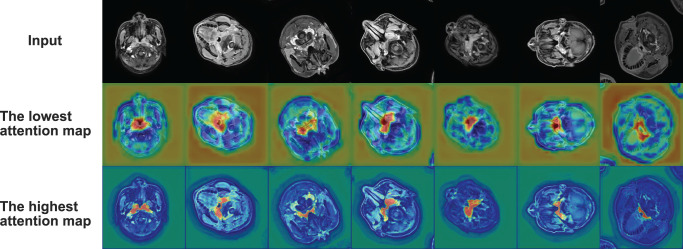
Radar plot showing the comparison of evaluation metrics obtained from each model in the comparison experiment. The five vertices of the radar plot respectively represent the average DSC value, average sensitivity, average specificity, average precision, and average Jaccard similarity coefficient. Note that this radar plot uses percentages to express the above evaluation metrics to make the comparison more distinguishable, as shown in equation 11.

(2) μi=ΨT(ReLU(λi))+bμi

(3) $$\alpha_{i}=\frac{1}{1+e^{-\mu_{i}}}$$

where *i* refers to the level of the encoder or decoder, Ψ*^T^* corresponds to a 1×1 convolution, λ*_i_* denotes the result of a linear transformation of input *X_e_*_(_ *_i_*_)_ and attention guidance *X_d_*_(_ *_i-_*_1)_, and *μ_i_* is the intermediate variable we define. Our model defines the attention coefficients as the output of a sigmoid layer *α_i_* and *α_i_* ϵ [0, 1]. According to the formula, our model utilizes more accurate semantic information from the higher level to guide the extraction of the attention region from the lower-level feature map. The output of the attention block is the attention coefficients and element-wise product of the input feature map. We visualize attention coefficients to the attention map, as shown in **Figure 3**.

Therefore, the output extracts the vital part of the input feature map for the segmentation while weakening its irrelevant part. In addition, our model uses soft attention. Its main feature is differentiable, making it easier to learn by backpropagation.

### 2.2 Recurrent Convolutional Block

The recurrent convolution is a commonly used method in text classification (39). Some studies apply it to the field of computer vision, such as Liang et al. (27) who use it in the Object Recognition and Alom et al. (40) who use it in the image segmentation. Inspired by the above studies, our model adds the recurrent convolution block to make it applicable for nasopharyngeal cancer segmentation.

In our model, recurrent convolutional layers replace standard convolutional layers so that our model can keep deepening and integrating contextual information on discrete time steps. It effectively improves the ability to extract detailed features. By setting the total time step parameter to *t*, the Conv+BN+ReLU operation repeats *t* times in each recurrent convolutional block. The input for each convolution is defined as follows:


(4) $$I(t)={\left(W_{f}\right)}^{T}X+{\left(W_{r}\right)}^{T}O(t-1)+b$$

showing that the input *I*(*t*) consists of the summation of the original input *X* and the output *O*(*t* – 1) of the previous convolution. *W_f_* and *Wr* refer to the weights of the feedforward path and recurrent path, and *b* is the bias. In addition, it is essential to note that *X* remains constant throughout the iteration.

In our Recurrent Convolutional Block, each loop contains the following operations:


(5) $$y_{1,\tau }={\left(W_{1,\tau }\right)}^{T}x+b_{1,\tau }$$

(6) $$y_{2,\tau ,i}=\gamma \times \frac{y_{1,\tau ,i}-\mu_{y_{1,\tau }}}{\sqrt{\sigma_{1,\tau }^{2}+e^{-5}}}+\beta$$

where *τ* refers to the current time step, *W*_1,_ *_τ_* refers to the weight of an ordinary convolution, *b*_1,_ *_τ_* refers to the bias, $\mu_{y_{1,\tau }}$ refers to the mini-batch mean of *y*_1,_ *_τ_*, $\sigma_{1,\tau }^{2}$ refers to its mini-batch variance, and *γ* and *β* are the two parameters to be learned. The second equation actually completes a batch normalization operation on *y*_1,_ *_τ_* .

In general, at each time step *t*, the model does a convolution on the input *I*(*t*). Then, the batch normalization (BN) and the activation function (ReLU) were applied to get the output *O*(*t*) of that time step and use it as one of the input terms for the next time step.

### 2.3 Residual Connection

Residual connection (26) is a well-known deep learning method. It represents the final output as a linear superposition of its input and a non-linear transformation of its original output. It combines them by direct summation. Many studies show that the application of residual connection in deep neural networks can effectively solve the network degradation problem (41, 42) and the shattering gradient problem (43) during the backpropagation of training. Besides, it can also make the training more accessible.

Let us assume that the mathematical representation of the residual connection of the *i*th layer in the *τ*-th time step is as:


(7) $$x_{i+1,\tau }=F[\alpha_{i,\tau }x_{i,\tau }+f({\left(W_{i,\tau }\right)}^{T}x_{i,\tau }+b_{i,\tau })]$$

where *x_i,τ_* is the input of the *i*th layer in the *τ*th time step, *α* refers to the modulating scalar, *W_i,τ_* refers to the weight of the recurrent convolution of the *i*-th layer in the *τ*-th time step, and *b_i,τ_* refers to its bias. *f* refers to the operation after recurrent convolution in each time step, which denotes the batch normalization and the ReLU activation function. *F* refers to the operation after each time step. Here, F is set to identity. Thus, when introducing the residual connection, our model defines a linear summation of the convolutional output and input after modulating as a new input for the next layer.

As the number of layers increases, the residual connections keep recurring; the residual connections on layer *I* relative to the previous layer *i* can be expressed as:


(8) $$x_{I,i,\tau }=(\prod_{k=i}^{I-1}\alpha_{k})x_{i,\tau }+\sum_{k=i}^{I-1}\theta_{I,k,\tau }$$

(9) $$\theta_{I,k,\tau }=(\prod_{h=k+1}^{I-1}\alpha_{h})f({\left(W_{k,\tau }\right)}^{T}x_{k,\tau }+b_{k,\tau })$$

where *x_i,τ_* refers to the input of the *i*-th layer in the *τ*-th time step, *α* refers to the modulating scalar, *W_k,τ_* refers to the weight of the recurrent convolution of the *k*th layer in the *τ*th time step, and *b_k,τ_* refers to its bias. *f* refers to the operation after recurrent convolution in each time step. Moreover, *θ_I,k,τ_* is the intermediate variable we define. Finally, *x_I,i,τ_* is the input of layer *I* with residual connections.

### 2.4 Normalization Method

Since the MRI data come from multiple batches of different MR scans, and there are differences in contrast agents, physician operations, etc., the brightness and contrast between images vary significantly. For example, the brightness of some images may be very dark, in which nasopharyngeal cancer appears as dark areas, while the brightness of other images may be very bright, with high contrast, in which nasopharyngeal cancer appears as bright areas, as shown in **Figure 1B**. It often requires precise adjustment during manual segmentation. In order to minimize the additional difficulties caused by data variation for training, our model normalizes all the input data.

Several previous studies also point that normalization can make the data more uniformly distributed. It can effectively improve the speed of solving the optimal solution by gradient descent, making it easier for the model to converge and potentially improve the model performance. Therefore, our model applies the Z-score normalization to all images based on all original pixels’ mean and standard deviation. It is also known as standardization. Images after processing have a mean of 0 and a variance of 1 conforming to the standard normal distribution.

### 2.5 Implementation Details

In the training process of AttR2U-Net, our model uses the Adam optimizer (44) to implement the gradient descent method and set the parameters *β*_1_ and *β*_2_ to 0.9 and 0.999, respectively. The learning rate is dynamically adjusted using the learning rate decay strategy. The model also uses L2 regularization (45), dropout (46), and other mechanisms to solve the overfitting problem to some extent. We train the model with the shuffled nasopharyngeal carcinoma images with segmentation labels. Moreover, we promptly test the model on the validation set at the end of each training epoch to adjust the parameters.

## 3 Experiment

### 3.1 Materials

#### 3.1.1 Data Acquisition

Our model uses MRI images from a total of 93 patients diagnosed with nasopharyngeal carcinoma. The patients are scanned by the Siemens Aera MRI system. The resulting MRI images are stored in Digital Imaging and Communications in Medicine (DICOM) file format. Specifically, all MRI data are T1-weighted, and contrast agents are used during the imaging process. The scanning area includes the entire head and neck region (approximately 100 slices), resulting in T1W+C images with a voxel spacing of 0.71875×0.71875×3mm^3^. We annotate the MRI images of each patient with the nasopharyngeal cancer boundary at the pixel level according to their pathological characteristics.

#### 3.1.2 Data Preprocessing

To utilize the raw data for 2D image segmentation, we extract 956 slices containing the nasopharyngeal cancer region from the DICOM files and transform them into 2D images. However, the raw images contain a large amount of black backgrounds. Our model crops each image to the region of interest (ROI) to reduce unnecessary computing workload. The cropped image includes the nasopharyngeal carcinoma area and the rest of the head region. Then our model resizes them to 256 × 256. Our model applies data augmentation methods, including rotation, mirroring, and affine transformation. In the end, our dataset includes 4,775 nasopharyngeal carcinoma images and their segmentation labels. 80% of the data are used for the training set, 10% are reserved as test set, and 10% are reserved as validation set.

### 3.2 Evaluation Method

In the training process, our model uses the most commonly used binary cross-entropy loss function, also known as BCELoss, to evaluate the training effect. The loss value *L* is defined as:


(10) $$L=-\frac{1}{N}\sum (y_{n}ln(p_{n})+(1-y_{n})ln(1-p_{n}))$$

where *N* refers to the total number of pixels, *y_n_* denotes whether the *n*th pixel belongs to the NPC region (if yes, then *y_n_* = 1), and *p_n_* denotes the probability that the pixel belongs to the NPC region according to our model’s prediction.

In the testing process, our model uses the test set of nasopharyngeal carcinoma MRI images (478 images in total) to evaluate the segmentation effect. To quantify the segmentation performance of our model, DSC value (47), Jaccard similarity (48), precision, specificity, sensitivity, and PR curve are used. In addition, we also perform patient-wise 5-fold cross validation experiments. We discuss our model performance in *Results*.

### 3.3 Comparison Method

In this study, we thoroughly review the field of computer vision and identify six advanced image segmentation models: SEUNet (49), FCDenseNet (50), NestedUNet (51), DeepLabV3 (52), DANet (35), and FCN (53). We use the official model of the corresponding papers and use the optimal parameters suggested by the authors. We then train them with the same training set for nasopharyngeal carcinoma segmentation and keep the same maximum number of training epochs for each model. For each comparison model, we select the best-performing trained model from the training process.

In the testing process, we use the same nasopharyngeal carcinoma segmentation test set to evaluate each model and calculate five evaluation metrics (the same as those used by AttR2U-Net) and plot PR curves. For demonstration, we also select seven typical MRI images of nasopharyngeal carcinoma as inputs and compare the output of each model, which are then compared with the manual segmentation result.

## 4 Results

### 4.1 Performance of Our Model

**Figure 4:**
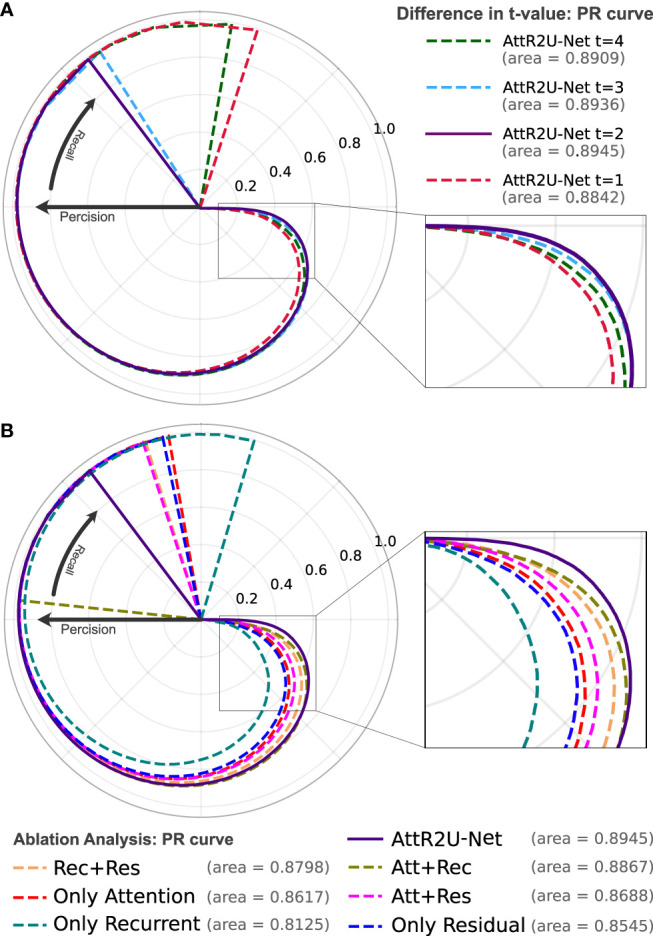
**(A)** The PR curve of AttR2U-Net with t values of 1, 2, 3, and 4. **(B)** The PR curve of the ablation analysis experiment includes 7 models with different parts of AttR2U-Net. The PR curves use a form of the rose diagram to show the comparison results more visually. The area value refers to the area under the PR curve in a plane right-angle coordinate system, also known as AUC. From **(A)**, our model achieves the best performance when t is taken as 2. From **(B)**, our model has the largest AUC value and is at the outermost part of the rose diagram.

**Figure 5:**
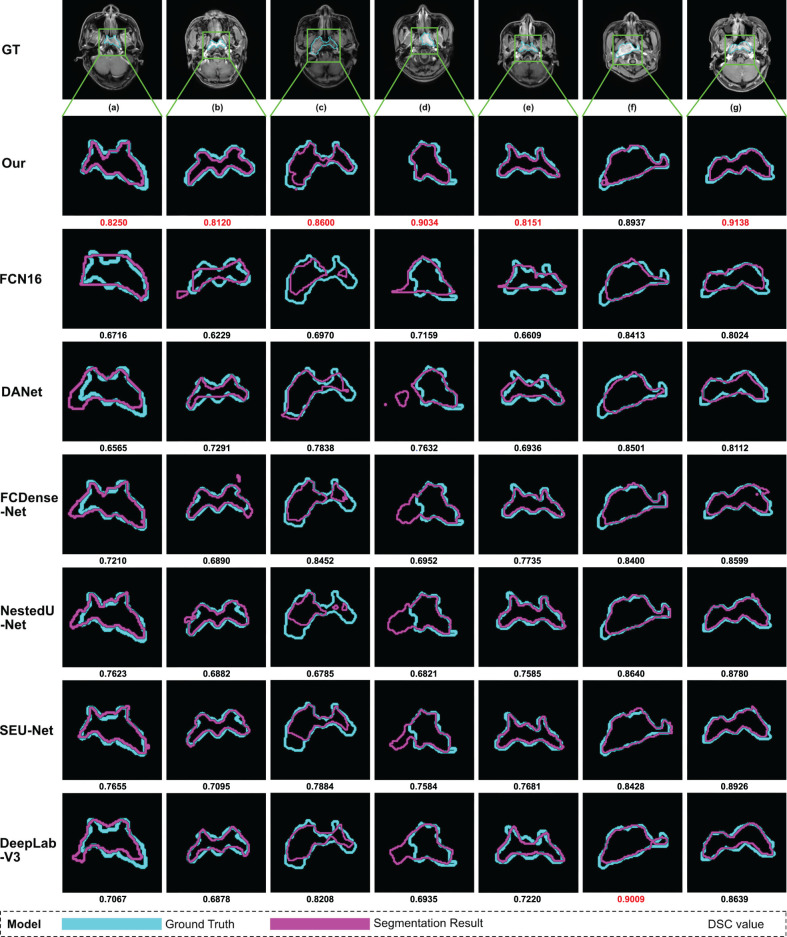
Examples of NPC segmentation results: We select seven typical MRI images of nasopharyngeal carcinoma and present the segmentation results of our model and seven models used for comparison. From top to bottom, the manual segmentation results and model segmentation results of our model, SEUNet, DANet, FCDenseNet, NestedUNet, DeepLabV3, and FCN16, respectively. For each MRI image of nasopharyngeal carcinoma, we draw the manual segmentation boundary with skyblue contours and the segmentation boundary of the corresponding model with purple contours and then calculate the DSC value, which is displayed below the image. We mark the maximum DSC value for each image in red.

The results show that our model is superior to other models for nasopharyngeal carcinoma segmentation. The test results of our model are as follows: The average DSC value obtained from the test is 0.816, the average Jaccard similarity is 0.692, the average precision is 0.825, the average specificity is 0.998, and the average sensitivity is 0.814. The area under the curve (AUC) in the PR curve (**Figure 4**, purple line) is 0.8945. We further compare the segmentation map output from the model with the ground truth obtained from manual segmentation in **Figure 5** (row 2). Our model achieves excellent segmentation results consistently across most nasopharyngeal cancer cases, where the segmentation target areas are large (**Figures 5C, F, G**) and small (**Figures 5A, B, E**). Our model also handles shape variations in nasopharyngeal carcinoma well. The performance is consistent across cases where nasopharyngeal carcinoma is more regularly shaped (**Figures 5D, F**) or less regularly shaped (**Figures 5B, E**). Overall, the segmentation results of our model overlap well with the manual segmentation ground truth.

We try to adjust the hyperparameter *t* of our model, where *t* refers to the number of loops in each recurrent convolution block. We set *t* to 1, 2, 3, and 4, respectively, and the comparison results are shown in **Table 1**. We also use PR curves to visualize the comparison, as shown in **Figure 4A**. From the above comparison, our model achieves the best performance at *t* = 3.

**Table 1:** Experiments indicate that our model achieves the best performance when t = 3.

t	DSC	JS	PC	SP	SE
**1**	0.792 ± 0.045	0.659 ± 0.061	0.722 ± 0.067	0.996 ± 0.001	0.885 ± 0.046
**2**	0.815 ± 0.039	0.690 ± 0.055	0.750 ± 0.057	0.997 ± 0.001	0.898 ± 0.045
**3**	0.816 ± 0.041	0.692 ± 0.058	0.825 ± 0.058	0.998 ± 0.001	0.814 ± 0.057
**4**	0.803 ± 0.046	0.675 ± 0.063	0.743 ± 0.072	0.997 ± 0.001	0.885 ± 0.046

### 4.2 Ablation Analysis

Ablation analysis aims to analyze the effectiveness of various components of AttR2U-Net in improving the segmentation accuracy of nasopharyngeal carcinoma. We compare the performance of eight variants of AttR2U-Net on the nasopharyngeal carcinoma segmentation task, including a variant only with spatial attention, a variant only with recurrent convolution, a variant only with residual connection, a variant with spatial attention and recurrent convolution, a variant with spatial attention and residual connection, a variant with recurrent convolution and residual connection, the skeleton, and the model without the normalization method.

The results shown in **Table 2** indicate that our intact model outperforms the eight comparison variants in several vital metrics, especially DSC value. Given the small differences in mean values for some metrics in **Table 2**, we perform a Kruskal–Wallis test, where the p-values obtained from testing each variant against our full model in terms of DSC values are 0.009 (Att+Rec), 2 × 10^–4^ (Att+Res), 7 × 10^–4^ (Rec+Res), 7 × 10^–6^ (Only Att), 1 × 10^–14^ (Only Rec), 3 × 10^–7^ (Only Res), 3 × 10^–7^ (Skeleton), 8 × 10^–21^ (Without Norm), indicating that each variant is significantly different from our full model on DSC values. We also plot PR curves for each ablation experiment as shown in (**Figure 4B**). Therefore, combining spatial attention, residual connection, recurrent convolution, and normalization method can improve the segmentation performance on the nasopharyngeal carcinoma to varying degrees. Specifically, spatial attention improves the accuracy of macroscopic localization, whereas residual connection and recurrent convolution improve segmentation boundary detail. Normalization solves the problem of contrast differences between MRI images.

**Table 2:** The ablation analysis validates the effectiveness of our model’s configuration.

Model	DSC	JS	PC	SP	SE
**Att+Rec+Res**	0.816 ± 0.041	0.692 ± 0.058	0.825 ± 0.058	0.998 ± 0.001	0.814 ± 0.057
**Att+Rec**	0.799 ± 0.038	0.668 ± 0.052	0.725 ± 0.050	0.996 ± 0.001	0.896 ± 0.047
**Att+Res**	0.785 ± 0.051	0.650 ± 0.067	0.780 ± 0.060	0.998 ± 0.001	0.803 ± 0.084
**Rec+Res**	0.792 ± 0.033	0.658 ± 0.045	0.723 ± 0.047	0.996 ± 0.001	0.879 ± 0.041
**Only Att**	0.783 ± 0.044	0.646 ± 0.058	0.736 ± 0.065	0.997 ± 0.001	0.846 ± 0.056
**Only Rec**	0.739 ± 0.058	0.592 ± 0.002	0.679 ± 0.079	0.996 ± 0.001	0.827 ± 0.073
**Only Res**	0.775 ± 0.048	0.636 ± 0.064	0.753 ± 0.059	0.997 ± 0.001	0.808 ± 0.077
**Skeleton**	0.769 ± 0.063	0.632 ± 0.080	0.713 ± 0.089	0.996 ± 0.002	0.858 ± 0.076
**Without Norm**	0.716 ± 0.060	0.562 ± 0.072	0.686 ± 0.071	0.996 ± 0.001	0.764 ± 0.097

### 4.3 Comparison With State-of-the-Art Models

We compare our model with six state-of-the-art models in a comprehensive manner. The comparison models include SEUNet, DANet, FCDenseNet, NestedUNet, DeepLabV3, and FCN16. Examples of NPC segmentation results for each model and their corresponding DSC values are shown in **Figure 5**. For cancerous regions with complex shapes, our model performs better in detail retention and has a higher degree of agreement with manual segmentation. For cases with abnormal cancerous location, our model can find the exact region more accurately than comparison.

We use the same five evaluation metrics as before to quantify the comparison results. Our model outperforms the above six comparison models in the critical metrics for nasopharyngeal carcinoma segmentation. Here we list the test results of the three models with the highest DSC values as follows: For NestedUnet, the average DSC value is 0.790±0.047, 3.19% lower than ours, the average Jaccard similarity is 0.657±0.063, 5.06% lower than ours, the average precision is 0.741±0.058, 10.18% lower than ours, the average specificity is 0.997±0.001, 0.10% lower than ours, and the average sensitivity is 0.856±0.064, 4.91% higher than ours. For DeepLabV3, the average DSC value is 0.787±0.040, 3.55% lower than ours, the average Jaccard similarity is 0.651±0.054, 5.92% lower than ours, the average precision is 0.706±0.057, 14.42% lower than ours, the average specificity is 0.996±0.001, 0.20% lower than ours, and the average sensitivity is 0.894±0.041, 8.95% higher than ours. For SEUNet, the average DSC value is 0.787±0.039, 3.55% lower than ours, the average Jaccard similarity is 0.651±0.053, 5.92% lower than ours, the average precision is 0.702±0.055, 14.91% lower than ours, the average specificity is 0.996±0.001, 0.20% lower than ours, and the average sensitivity is 0.903±0.043, 9.86% higher than ours.

**Figure 6:**
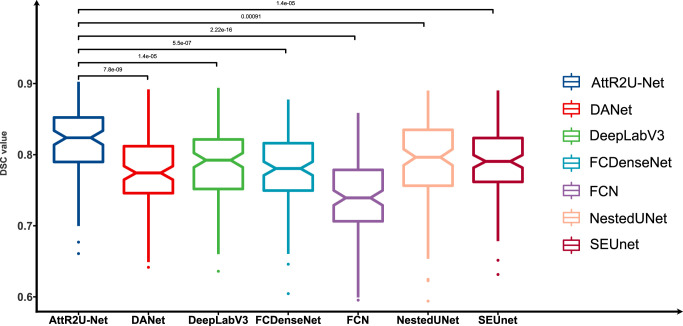
Box plots of the DSC values obtained from the tests on the test set and its Kruskal-Walis test results. The results show that our model achieves the highest DSC values in the test of nasopharyngeal carcinoma segmentation and is significantly different from other models.

Given the small differences in the mean values of certain metrics in the model comparisons, we perform the Kruskal–Wallis test, in which the p-values obtained from testing each model against our model on DSC values are 0.001 (NestedUnet), 1.4 ×10^–5^ (DeepLabV3), 1.4 ×10^–5^ (SEUnet), etc., indicating that each model is significantly different from our model on DSC values. The box plots of DSC values obtained from the tests of each comparison model and the Kruskal–Walis test results are shown in **Figure 6**.

**Figure 7:**
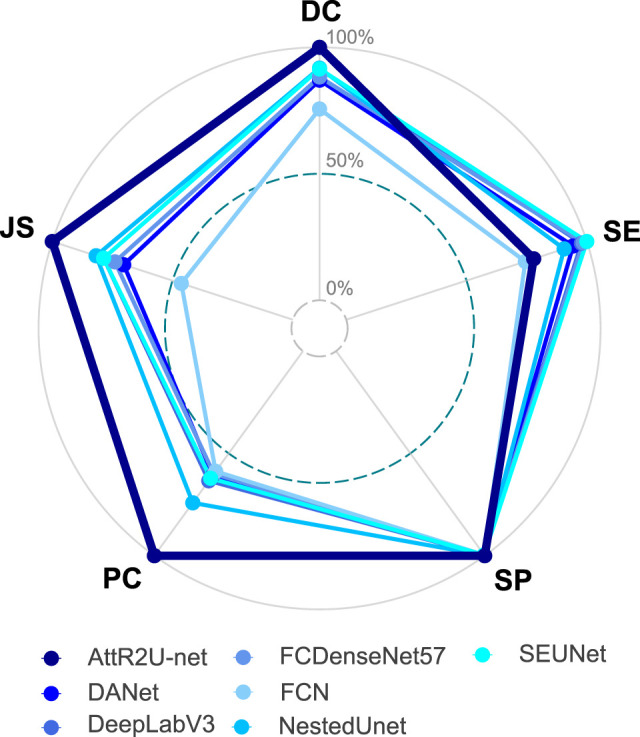
Radar plot showing the comparison of evaluation metrics obtained from each model in the comparison experiment, which includes our model, DANet, DeepLabV3, FCDenseNet, FCN, NestedUNet, and SEUNet. The five vertices of the radar plot respectively represent the average DSC value, average sensitivity, average specificity, average precision, and average Jaccard similarity coefficient. Note that this radar plot uses percentages to express the above evaluation metrics to make the comparison more distinguishable, as shown in equation 11.

**Figure 8:**
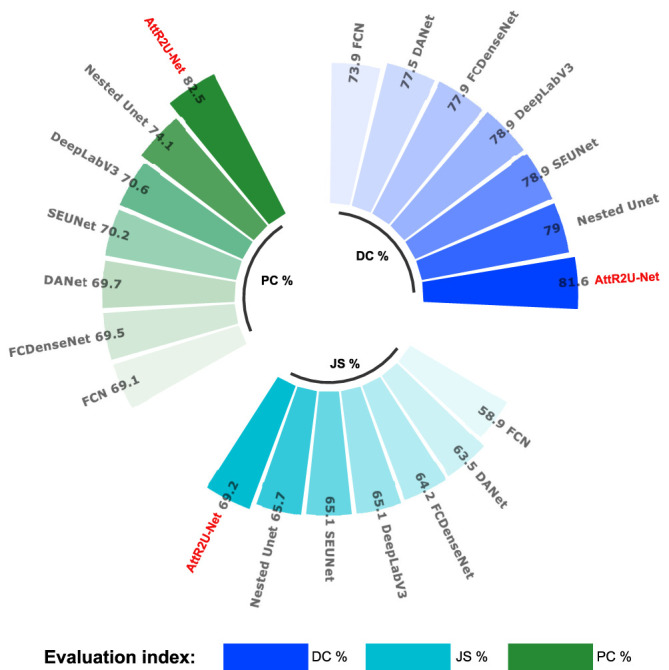
Comparison plots of specific values of 3 key evaluation metrics: include average DSC values, average Jaccard similarity coefficient, and average precision obtained from the test on the nasopharyngeal cancer segmentation test set.

To show the performance differentiation among the models more intuitively, we plot testing results as radar plots shown in **Figure 7**. Additionally, the radar plot transforms the values of each metric into percentage form to make 0.5 value 0% and the maximum value 100%. The conversion formula is as:


(11) Yi=Xi−0.5Maxi−0.5×100%

where *Max_i_* denotes the maximum value of the *i*th evaluation metric, *X_i_* denotes the test result of the model on that evaluation metric. For the radar plots, the more peripheral the hexagon enclosed by the model is, the better the model’s performance is. The hexagon enclosed by our model is at the outermost part. Moreover, our model ranks first in all evaluation metrics except for a slightly lower SE value. In addition, we plot the specific values of 3 key metrics (DSC value, Jaccard similarity, and precision) for each model, as shown in **Figure 8**. Our model achieves the maximum value on these three most widely used evaluation metrics.

**Figure 9:**
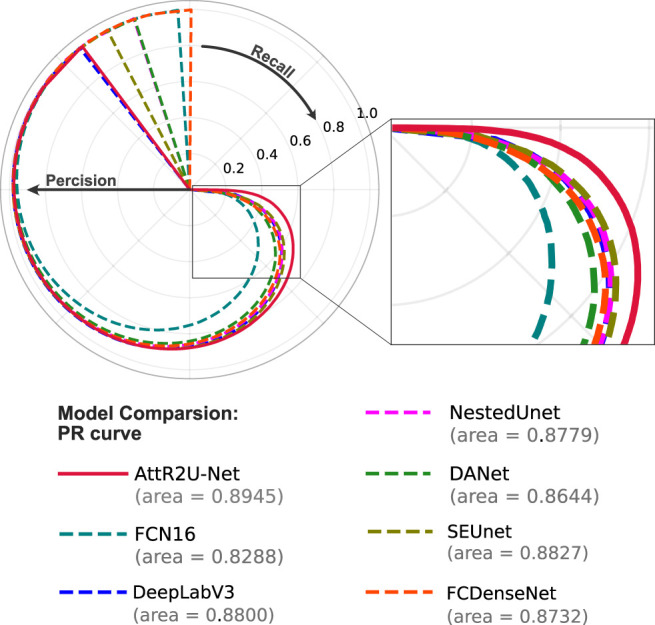
The PR curve of model comparison on the nasopharyngeal carcinoma segmentation test set. The area value indicates the AUC value of each PR curve, and it can be seen that the AttR2U-Net model has the highest AUC value and is located at the outermost part.

PR curves shown in **Figure 9** compare our model with the state-of-the-art model. Among the seven models, our model has the largest area under the curve (AUC). The four models with the best AUC are AttR2U-Net (AUC = 0.8945), SEUnet (AUC = 0.8827, 1.32% lower than ours), DeepLabV3 (AUC = 0.8800, 1.62% lower than ours), and NestedUnet (AUC = 0.8779, 1.86% lower than ours).

To further demonstrate our model’s generalization performance, we also perform the patient-wise 5-fold cross validation experiments. **Table 3** presents the model comparison results (DSC value) for the patient-wise 5-fold cross validation. Our model achieves a mean DSC value of 0.7914±0.021 (0.7078~0.7914) in cross-validation. The number of nasopharyngeal carcinoma slices included in each patient case varies widely, ranging from a few to dozens, resulting in different sizes of the training set and test set between different fold numbers. Thus, the results of each experiment in the patient-wise 5-fold cross validation are somewhat different.

**Table 3:** Results (DSC value) of model comparison experiments using patient-wise 5-fold cross validation.

Model	Fold number					Mean
	1	2	3	4	5	
**AttR2U-Net**	0.8020	0.8044	0.8030	0.7552	0.7923	0.7914 ± 0.021
**Nested UNet**	0.8080	0.7870	0.8023	0.7295	0.7627	0.7779 ± 0.032
**SEUnet**	0.8118	0.7893	0.7926	0.7353	0.7588	0.7776 ± 0.030
**FCDenseNet**	0.7871	0.7561	0.7932	0.7327	0.7126	0.7563 ± 0.035
**DeepLabV3**	0.7722	0.7435	0.7708	0.7059	0.7499	0.7485 ± 0.027
**DANet**	0.7651	0.7269	0.7474	0.6933	0.7146	0.7295 ± 0.028
**FCN**	0.7461	0.6885	0.7247	0.6727	0.7072	0.7078 ± 0.029

The above results suggest that our model is the most reliable for the fully automated end-to-end nasopharyngeal cancer segmentation task. For nasopharyngeal carcinoma, which is relatively complex and difficult to segment accurately, the AttR2U-Net structure could achieve better performance in terms of accurate localization and detail preservation of the cancerous region.

## 5 Discussion

This study designed a novel model for automatic nasopharyngeal carcinoma segmentation called AttR2U-Net featuring spatial attention, residual connection, recurrent convolution, and the normalization method. The combination of these features dramatically improves the segmentation performance, allowing our model to take a closer step to the full automation of the nasopharyngeal carcinoma area segmentation. Accurate segmentation of the nasopharyngeal carcinoma area is critical to patients’ radiotherapy. Our end-to-end AttR2U-Net model can efficiently segment the nasopharyngeal carcinoma region in a certain degree of accuracy, significantly saving specialized physicians valuable time and circumventing the boundary variances caused by different physicians. We demonstrate that our model is more robust to irregular shapes of nasopharyngeal carcinoma than state-of-the-art models in the literature.

In previous studies of lesion segmentation, deep learning and artificial intelligence techniques are widely used in relatively simple tasks such as segmentation of organs like lung (54), liver (55), and ventricular (56), pancreas (57). Unlike the segmentation in the organs above, the segmentation of nasopharyngeal carcinoma has a higher complexity. It is because its location is in the brain, and the tissue structure in the human brain is the most complex in the human body. The differentiation between nasopharyngeal carcinoma and the surrounding tissues is extremely challenging as its cancerous area is usually mixed with surrounding tissues. Even a professional doctor takes a long time to build one patient’s manual segmentation. In addition, compared with other computer vision tasks (58, 59), extremely high precision and detail preservation hold higher stakes for medical image segmentation, as inaccuracies in NPC segmentation may lead to damages of the patient’s brain.

Our model achieves a DSC value, a commonly applied measure of the similarity of segmentation results to ground truth, of 0.816 on the nasopharyngeal carcinoma segmentation task. In this study, we compare AttR2U-Net with six other advanced image segmentation models and do ablation analysis experiments. To the best of our knowledge, our model achieves the highest level of performance among fully automated nasopharyngeal carcinoma segmentation models. Specifically, our model design performs more precise localization and higher detail preservation of nasopharyngeal cancer segmentation. As such, our model has significant practical implications for the NPC treatment.

However, our model still has a small number of outlier cases with poor segmentation, mainly those nasopharyngeal carcinomas with a large extent, highly unconventional shape, and extremely remote positions. Our model may be used to assist physicians in segmenting the cancerous region. Nevertheless, given the importance of this segmentation for radiotherapy, it still cannot perform the segmentation task thoroughly in a fully standalone manner. The physician’s post-check and calibration remain necessary.

## 6 Conclusion

This study proposed a fully automated end-to-end segmentation model called AttR2U-Net for nasopharyngeal cancer segmentation. Our model creatively combines several advanced computer vision methods, including spatial attention, residual connection, recurrent convolution, and normalization. Compared with other state-of-the-art image segmentation models, our model has the highest segmentation performance. Additionally, our model has excellent efficiency relative to manual segmentation. Thus, it is a promising model to assist physicians in the radiotherapy of nasopharyngeal carcinoma.

## Data Availability Statement

The datasets presented in this article are not readily available because the data are not publicly available due to data confidentiality. Requests to access the datasets should be directed to liuxj86@mail.sysu.edu.cn.

## Ethics Statement

Ethical review and approval were not required for the study on human participants in accordance with the local legislation and institutional requirements. Written informed consent for participation was not required for this study in accordance with the national legislation and the institutional requirements.

## Author Contributions

JZ: Conceptualization, Methodology, Software, Validation, Writing - Original Draft. LG: Validation, Writing – Review & Editing, Supervision. GH: Conceptualization, Validation, Writing – Review & Editing, Supervision. XL: Conceptualization, Validation, Writing – Review & Editing, Supervision.

## Funding

This work was supported by the National Natural Science Foundation of China (61901533) and the Shenzhen Fundamental Research Program, China (JCYJ20190807154601663).

## Conflict of Interest

The authors declare that the research was conducted in the absence of any commercial or financial relationships that could be construed as a potential conflict of interest.

## Publisher’s Note

All claims expressed in this article are solely those of the authors and do not necessarily represent those of their affiliated organizations, or those of the publisher, the editors and the reviewers. Any product that may be evaluated in this article, or claim that may be made by its manufacturer, is not guaranteed or endorsed by the publisher.
